# Minimally Invasive Excision of a Giant Paratubal Cyst: Case Report and Management Review

**DOI:** 10.1155/2019/3458230

**Published:** 2019-12-23

**Authors:** Bassem Skaff, Dani Zoorob, Rawane El Assaad, Mohamad Abou-Baker

**Affiliations:** ^1^Nini Hospital, Achier El Daya Street, Tripoli, Lebanon; ^2^Department of Obstetrics and Gynecology Academic Offices, University of Toledo, 2142 N. Cove Blvd., Toledo, OH 43606, USA

## Abstract

Paratubal cysts are usually incidentally found due to being small and asymptomatic adnexal structures. Enlargement to more than 15 cm concurrent with late presentation in an adult is rare. We present the case of a 36 cm diameter cyst in a 31-year-old female whose symptoms involved overactive bladder, abdominal bloating, and pressure. Radiologic findings suggested that it extended from the xiphoid and pubic symphysis. Removal was through a minimally invasive technique.

## 1. Introduction

A paratubal cyst is a closed, fluid-filled sac that grows near the ovary and often connected to the fallopian tube. Paratubal or paraovarian cysts represent approximately 10% of all adnexal masses, often unilateral, and benign. They usually originate from the mesothelial covering of the peritoneum or from paramesonephric and mesonephric remnants. Thus, they are covered by a single layer of ciliated columnar or flattened cells [1]. These cysts are in most cases asymptomatic and noted as incidental findings during other pelvic examinations or surgery. Whereas in most cases they are measured between 2 and 20 mm, few cases are reported to exceed 15 cm in diameter.

In this case, we describe the unique case of a giant paratubal cyst measuring 36 cm in diameter excised in a minimally invasive technique.

## 2. Clinical Case

**Figure 1:**
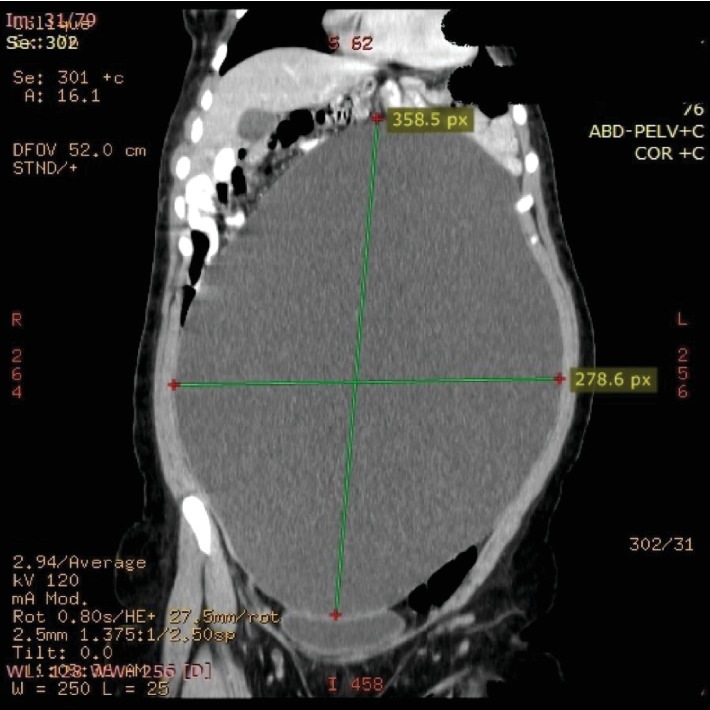
CT scan demonstrating the largest dimensions of the cyst (36 × 28 cm) prior to excision.

**Figure 2:**
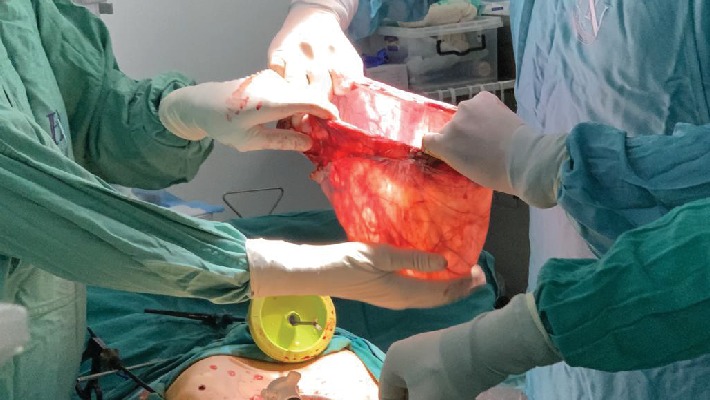
The paratubal cyst sac size noted following excision.

A 31-year-old woman presented with abdominal distention and discomfort worsening over the past 6 months. Concurrently, she had development overactive bladder symptoms, bloating, and dyspepsia. Her medical history was pertinent for epilepsy controlled with antiepileptic medications. Urine analysis was negative for any signs of infection, and her pregnancy test was negative. Abdominal examination was positive for a firm and distended abdomen, uniformly enlarged from the xiphoid bone to the pubic symphysis. No tenderness was reported on palpation; however, pressure was endorsed. A bedside abdominal ultrasound was performed that revealed an abdomen fully distended with fluid, initially suspected to be ascites. Computed tomography in 2.5 mm helical slices with both IV and oral contrast suggested the presence of an encapsulated cystic mass containing clear fluid (6 Hounsfield units). Dimensions reported were 36 cm in long axis, 28 cm in transverse diameter, and 15 cm in anteroposterior diameter, corresponding to 7 liters in volume, suggestive of a right ovarian cystadenoma (Figures 1 and 2). No capsular irregularities were seen or intracystic vegetations identified. Concurrently noted was right renal pelvicalyceal dilatation resulting from compression due to the proximity of the right ureter with the renal pelvis measuring 2 cm in anteroposterior diameter. No abdominal ascites or other abdominopelvic abnormalities noted. Tumor markers were in the normal ranges (CA125: 12.13 U/ml, CEA:1.51 ng/ml, and AFP: 1.46 IU/ml). While understanding her risks, the patient stressed the avoidance of excision of ovarian or fallopian tissue due to fertility concerns and preferred that the cyst be removed in a cosmetic manner including avoidance of larger laparotomy incisions.

**Figure 3:**
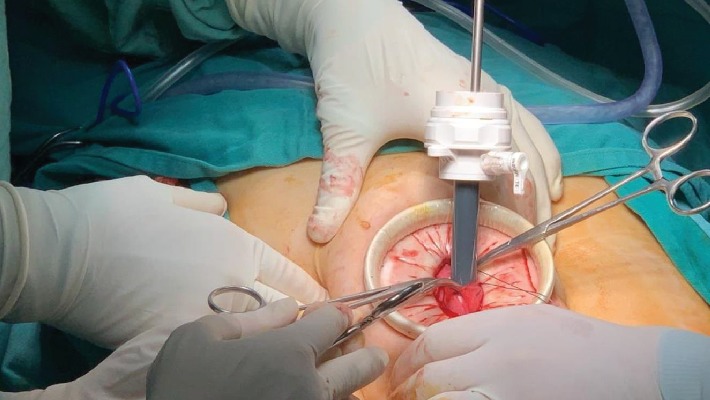
The figure demonstrates the 11 mm trocar introduced into the cyst cavity to facilitate drainage. A purse-string suture placed around the trocar is also demonstrated. A 5 mm suction tube was used to drain the cystic fluid contents through the trocar.

The patient underwent resection of the mass. The procedure was begun by drainage of the cyst in order to identify the stalk and excise all cyst walls. A 3 cm supraumbilical incision was performed with careful dissection until reaching the peritoneal cavity. An Alexis O™ wound protector/retractor Applied Medical was introduced through the small incision permitting better visualization of the cyst (Figure 3). Three hemostats were used to hold the cyst wall in place, followed by meticulous introduction of an 11 mm trocar into the cyst cavity under direct vision. This was followed by insertion of a 5 mm suction tube through the trocar to aspirate the fluid contents while avoiding spillage of fluid into the peritoneal cavity. Eight liters of serous fluid were aspirated. Following fluid evacuation, the cyst wall had collapsed. The trocar site in the cyst wall was sutured shut in a purse-string manner followed by a second closure layer. Then, the cyst was released off the hemostats, and the laparoscopic gel cap was fitted onto the Alexis retractor to convert the abdominal incision into a laparoscopic port site. This was used to insufflate the abdomen with CO_2_. Thereafter, 2 additional 5 mm trocars on each side of the midline were introduced.

**Figure 4:**
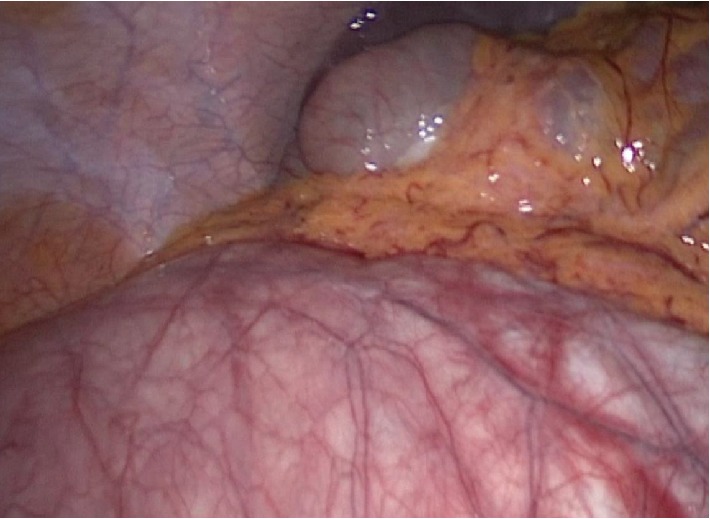
The giant paratubal cyst sac after drainage persistently reaching the upper abdominal structures (the liver and the gall bladder).

The cyst borders were identified (Figure 4). Using the bipolar forceps and scissors, it was completely dissected off the fallopian tube. Both the fallopian tube and ovarian tissues were noted to be intact. The cyst was removed through the supraumbilical incision and sent to pathology. The trocars were removed and all incisions approximated as per protocol. The patient was discharged soon thereafter from the observation unit.

## 3. Discussion

In our case, we discuss the option of management of a giant paratubal cyst using a minimally invasive technique. Few documented cases report sizes comparable to our patient's case, and thus, management had to be individualized. Furthermore, larger cysts pose a conundrum as there is a concern for malignancy, adequate removal demands, and appropriate excision, ensuring preservation of fertility of certain populations, as well as attempting to offer treatment with the least risk to the patient.

Of the larger paratubal cysts reported, sizes have reached 30 cm maximally whereas our patient had a cyst measuring 36 cm in diameter [2, 3]. Fluid content reported has varied per case, but similar to ours, most procedures proceeded with drainage of the high volume prior to excision of the cyst. Similarly, presentation for the larger cysts has been reported to have concurrent resultant renal pathology extrinsic to the kidney itself. Similar to the case by Leanza et al. whose patient had hydronephrosis, our patient also had renal involvement detected by imaging and noted to be marked pelvicalyceal dilation [2].

Paratubal cysts arise from embryologic remnants located in the proximity of the broad ligament between the ovary and the fallopian tube. Incidence rates are reported to be 17% to 33% for patients diagnosed with benign adnexal cysts [4]. Smaller paratubal cysts are much more frequently encountered in women during the third and fourth decades of life compared to children and adolescents, whereas larger ones present earlier in life. Symptomatic complications include hemorrhage and torsion, with the risk being greater once cyst diameter exceeds 5 cm. Giant paratubal cysts larger than 10 cm in diameter are rare with few reports in the literature especially when presentation in later in life [1, 5].

Ultrasound and CT imaging in this patient helped in the diagnosis and guided the management permitting a minimally invasive approach. However, the definitive diagnosis requires surgical exploration to confirm cyst origin and its pathology. Ultrasound findings that should raise the clinician's level of concern regarding malignancy include cyst size greater than 10 cm, papillary or solid components, cyst wall irregularities, presence of ascites, and intratumoral high-color Doppler flow. There has been significant research on the use of ultrasound scoring systems alone or in combination with serum markers or historical information for predicting malignancy [7, 8]. Serum marker testing is indicated to evaluate the likelihood of malignancy and concurrent management. Elevated CA 125 levels in combination with other findings may be useful to distinguish between benign and malignant adnexal masses and to identify patients who should be treated by a gynecologic oncologist. Low specificity occurs premenopausally because the CA 125 level is elevated in many nonmalignant clinical conditions and inflammatory conditions [6]. Calculators for malignancy risk have been described that help with stratifying risk [9]. Specificity and positive predictive value of CA 125 levels are consistently higher in postmenopausal women compared with premenopausal women [8, 10]. The overall sensitivity of CA 125 testing in distinguishing benign from malignant adnexal masses reportedly ranges from 61% to 90%; specificity ranges from 71% to 93%, positive predictive value ranges from 35% to 91%, and negative predictive value ranges from 67% to 90% [8, 10–12].

Similar to Salem and Vecchio et al., resolution of the cyst was through a minimally invasive technique [3, 13]. Midline laparotomy was avoided as the likelihood of malignancy was low based on prior testing. Thus, despite the giant size of the cyst, patient management was possible through small incisions thus permitting for rapid recovery, faster return to daily life activities, and reducing risk of postoperative complications. The options of use of robotics may be possible for management of such cases although conventional laparoscopy offers similar outcomes at lower cost. Furthermore, conventional laparoscopy is preferred because of its shorter operative time [14].

## 4. Conclusion

This case report delineates the management of one of the largest paratubal cysts reported while offering the patient a safe minimally invasive treatment modality. Accordingly, management of these cysts is possible once malignancy has been ruled out. Imaging and laboratory testing help guide the preoperative planning.

## References

[1] Cevrioglu A. S., Polat C., Fenkci V., Yilmazer M., Yilmaz S., Dilek O. N. (2004). Laparoscopic management following ultrasonographic-guided drainage in a patient with giant paraovarian cyst. *Surgical Endoscopy*.

[2] Leanza V., Coco L., Genovese F. (2013). Laparoscopic removal of a giant paratubal cyst complicated by hydronephrosis. *Il Giornale di Chirurgia*.

[3] Vecchio R., Leanza V., Genovese F., Accardi M., Gelardi V., Intagliata E. (2009). Conservative laparoscopic treatment of a benign giant ovarian cyst in a young woman. *Journal of Laparoendoscopic & Advanced Surgical Techniques*.

[4] Okada T., Yoshida H., Matsunaga T. (2002). Paraovarian cyst with torsion in children. *Journal of Pediatric Surgery*.

[5] Letourneur B., Grandjean S., Richard P., Parant O. (2006). Management of a giant paraovarian cyst. *Gynécologie, Obstétrique & Fertilité*.

[6] Ghaemmaghami F., Karimi Zarchi M., Hamedi B. (2007). High levels of CA125 (over 1,000 IU/ml) in patients with gynecologic disease and no malignant conditions: three cases and literature review. *Archives of Gynecology and Obstetrics*.

[7] Abbas A. M. (2015). The predictive value of transvaginal color and pulsed Doppler in evaluation of adnexal masses. *Thai Journal of Obstetrics and Gynaecology*.

[8] Abbas A. M., Zahran K. M., Nasr A., Kamel H. S. (2014). A new scoring model for characterization of adnexal masses based on two-dimensional gray-scale and colour Doppler sonographic features. *Facts, Views & Vision in ObGyn*.

[9] Woolas R., Jacobs I., Davies A. P. (1994). *What is the true incidence of primary fallopian tube carcinoma?*. *International Journal of Gynecological Cancer*.

[10] Abbas A. M., Amin M. T. (2015). Brenner's tumor associated with ovarian mucinous cystadenoma reaching a huge size in postmenopausal woman. *Journal of Cancer Research and Therapeutics*.

[11] Antonic J., Rakar S. (1996). Validity of colour and pulsed Doppler US and tumour marker CA 125 in differentiation between benign and malignant ovarian masses. *European Journal of Gynaecological Oncology*.

[12] Sehouli J., Akdogan Z., Heinze T. (2003). Preoperative determination of CASA (cancer associated serum antigen) and CA-125 for the discrimination between benign and malignant pelvic tumor mass: a prospective study. *Anticancer Research*.

[13] Salem H. A. (2002). Laparoscopic excision of large ovarian cysts. *The Journal of Obstetrics and Gynaecology Research*.

[14] El Khouly N. I., Barr R. L., Kim B. B. (2014). Comparison of robotic-assisted and conventional laparoscopy in the management of adnexal masses. *Journal of Minimally Invasive Gynecology*.

